# Barriers to Professional Help-seeking for Distress and Potential Utility of a Mental Health App Components: Stakeholder Perspectives

**DOI:** 10.7759/cureus.7128

**Published:** 2020-02-28

**Authors:** Jemimah A Johnson, Janhavi Devdutt, Seema Mehrotra, Poornima Bhola, Paulomi Sudhir, Amit Sharma

**Affiliations:** 1 Clinical Psychology, National Institute of Mental Health and Neurosciences, Bengaluru, IND; 2 Technology for Emerging Markets, Microsoft Research India, Bengaluru, IND

**Keywords:** mental health apps, barriers, help-seeking, help-seeking for distress, help-seeking intervention, common mental disorders

## Abstract

Introduction

A high prevalence of common mental disorders and the associated treatment gap, particularly in low and middle-income countries such as India, calls for novel mental health approaches with widespread reach. There is a need to enhance our understanding of the barriers experienced by distressed persons as well as to utilize these insights for breaking such barriers. Despite the rise in the use of technology-based solutions in the field of mental health, there is a dearth of app-based interventions that help in breaking barriers to seeking professional help for mental health concerns in distressed persons. The present study aimed at exploring the perspectives of distressed persons concerning barriers to seeking professional help for mental health concerns. It also sought to understand their perspectives on the perceived utility of proposed app components for breaking these barriers.

Methods

The study utilized a cross-sectional exploratory design. The sample included two groups of distressed participants who could be considered potential users (and thereby the stakeholders) of a mental health app under development for common mental health concerns: distressed treatment seekers (D-TS) and distressed non-treatment seekers (D-NTS). The D-TS group included 10 individuals (average age: 33 years; six men) with self-reported or clinician-reported depressive and anxiety symptoms at intake who were seeking help from mental health professionals. The D-NTS group included 10 distressed individuals (average age: 23 years; five men) who were recruited from the local community through an announcement. The announcement called for participants who were experiencing anxiety and low mood but had not yet sought help for their distress. A semistructured interview schedule was used to explore the nature of barriers encountered and the perceived utility of the content of the proposed app. The questions that aimed at understanding the perceived barriers were open-ended. The perceived utility of various components of the proposed app was explored via 11 items, with a 5-point Likert scale.

Results

Personal barriers frequently reported by both groups were doubts about treatment and fear of social consequences. The role of inadequate self-awareness about one’s mental health concerns as a barrier to reaching out for professional help was articulated more frequently by the D-TS group than the D-NTS group. Proposed app components such as self-assessment with individualized feedback, informative videos by mental health professionals, testimonials from mental health service users, and a platform for an online connection with a professional were rated as potentially useful in reducing barriers to professional help-seeking. Insights based on stakeholder perspectives have implications for further research and are being utilized for the development of a mental health app for common mental health concerns.

## Introduction

According to the National Mental Health Survey of India (2015-16), common mental disorders like depression and anxiety are prevalent and burdensome, affecting 10% of the population [1]. Alongside this alarming statistic, a wide gap exists between the prevalence of mental health problems and available treatments/services on the one hand and the number of individuals accessing these mental health services on the other. Studies indicate that the treatment gap is as high as 50% in most countries; this can go up to 90% in low-resource countries [2]. A large-scale epidemiological survey in India showed that only five among 100 individuals with any common mental health disorders received treatment, making the treatment gap as high as 95% [3]. One of the reasons for this treatment gap is the low contact coverage (i.e., the coverage of people who use the services or are in contact with a mental healthcare provider). Contact coverage is as low as 12% among people with common mental health disorders in India [4]. This underscores the need to explore reasons as to why distressed individuals do not seek help from mental health services or professionals.

Barriers that prevent people from seeking help for mental health concerns are manifold. Broadly, these include stigma-related, attitudinal, and instrumental barriers [5]. Some specific barriers to professional help-seeking for mental health concerns include stigma, unavailability of resources to seek help, lack of awareness about mental health or how to seek help, lack of support from others to seek help, and fear about the intervention or treatment [5,6]. Some of the other, albeit infrequently reported, barriers include financial and time constraints and fears about the side effects of medication. A scoping review indicated that treatment-related stigma had a negative effect on help-seeking, and issues related to disclosure were a common concern [7]. A National Comorbidity Survey in the United States revealed that self-reliance emerged as a major reason to drop out of treatment or to not seek help [8].

Global reports suggest that the treatment gap can be reduced by increasing the number of trained mental health professionals available for service delivery, the involvement of non-specialist care providers in mental health settings, and the active participation of people affected by mental disorders [2]. This is particularly true for low- and middle-income countries such as India. This points to the importance of taking the stakeholders’ perspective into account while devising mental health interventions. A review of published Indian literature indicates a dearth of studies that look into self-perceived barriers to help-seeking in distressed persons. Hence, the present study attempted to explore perceived barriers in two potential groups-distressed persons who are currently seeking help: distressed-treatment seekers (D-TS) and distressed persons who are not seeking help (distressed-non-treatment seekers, D-NTS). We expect that these findings would be of use in understanding perceptions of barriers and enablers across the continuum of the help-seeking process.

The increased use of technology alongside the widespread treatment gap has led researchers to explore the use of digital media for mental health engagement. The use of digital platforms has been considered a “game-changer” in combating these barriers and promoting mental health awareness [9]. However, there are few empirical studies related to the perceived utility or uptake of digital mental health platforms [10]. Additionally, although there is a voluminous research literature on internet-based and mobile-based applications for common mental health problems, most of these are based in the western context. Mobile platforms have a wide reach and can play an important role in mental health promotion in South-East Asia [11]. The majority of mobile apps are aimed towards increasing awareness by providing information on mental health conditions or suggesting self-help strategies to users for managing their mental health concerns [12]. There are very few empirical studies on preventive and promotive interventions using mobile apps, and their potential to reduce barriers to help-seeking in distressed persons has not been sufficiently explored [10].

Against this backdrop, the authors have engaged in a larger study aimed towards the development of a mental health app for persons experiencing common mental concerns. This app is expected to help users understand the nature of their psychological distress, explore self-help strategies for managing distress, and, at the same time, encourage them to connect with mental health professionals when needed. It proposes to achieve the latter by including components in the app that are specifically aimed at reducing barriers to help-seeking from mental health professionals. The current paper presents findings from one segment of this larger study. The twin aims of the present study were: 1) to understand perceived barriers to seeking professional help in a sample of potential users of a mental health app, and 2) to understand the perspectives of potential users of the mental health app about the perceived utility of the proposed app components for breaking barriers to seek help from a mental health professional.

## Materials and methods

The study adopted a cross-sectional exploratory design involving both quantitative and qualitative methods. The sample included two groups of participants who could be considered potential users (and thereby the stakeholders) of a mental health app for common mental health concerns.

The D-TS group included 10 individuals (average age: 33 years; six men) with self-/clinician-reported depressive and anxiety symptoms at intake, and who were seeking help from mental health professionals. These participants were recruited from the outpatient mental health services of a large tertiary mental health Institute in southern India and a community-based well-being center of the same institute.

The D-NTS group included 10 distressed individuals (average age: 23 years; five men). They responded to an announcement (flyer) that was circulated for one month through social media platforms, websites, and local colleges. It called for participants who were experiencing anxiety and low mood but had not yet sought help for their distress.

For both groups, the common criteria for recruitment included the following: age of 18 years and above, fluency in spoken and written English, and access to a smartphone.

To examine the nature of barriers and the perceived utility of the content of the proposed app, a semistructured interview schedule was prepared by the authors. The interview schedules for both the groups were similar except for a few probes. Probes for the D-TS group included barriers encountered during the process of reaching out for professional help, facilitators during the process of reaching out for professional help when they became aware of their mental health needs, and barriers faced during the actual utilization of mental health services. Probes for the D-NTS group included barriers that prevented them from seeking professional help for their current distress and potential motivators or facilitators to reach out for professional help.

The questions that aimed at understanding the perceived barriers were open-ended. The perceived utility of the content of the proposed app was explored through closed-ended questions that consisted of 11 items. These questions were common to both groups. Across these 11 items, the participants used a 5-point Likert scale to indicate the usefulness of the proposed content for breaking barriers to seeking professional mental health help. These items, reflecting various potential content components, were based on brainstorming within the research team, the clinical experience of the members, as well as existing relevant literature on professional help-seeking for mental health.

The study was reviewed and approved by the Institute Ethics Committee of the National Institute of Mental Health and Neurosciences, Bengaluru, and written informed consent was obtained from all participants. Participants in the D-TS group were recruited from the outpatient mental health services of a large tertiary mental health institute in southern India and a community-based well-being center of the institute, with self- or clinician-reported depressive or anxiety symptoms. For the D-TS group, written informed consent was obtained before conducting face-to-face interviews. The D-NTS group was comprised of individuals who expressed their willingness to participate in response to online announcements. In all, 12 individuals responded to the announcement. One of the individuals did not get back to the researchers after his initial expression of interest despite their attempts to re-establish contact. The other 11 individuals were educated by phone about the nature of the study. Following this, an informed consent form was sent to them via email. The first 10 responders who provided informed consent were recruited. One of the individuals gave consent but did not respond to the researchers thereafter. An email was sent to her notifying her of the completion of the recruitment process. The D-NTS group was given the option to either make time for a telephone interview or respond to the same questions via an online survey. Of the 10 participants, nine participated in a phone interview while one responded to the online survey. All the participants were debriefed after their interview about the barriers they expressed. Also, the D-NTS group was encouraged to seek help from mental health services near their location. They were provided referral-related guidance if required.

Participant responses were recorded manually by the researchers. Content analysis was carried out to delineate themes related to perceived barriers. The themes were identified jointly by the first (JAJ) and the second (JD) authors. The broader grouping of themes was arrived at through a joint discussion between the first three authors (JAJ, JD, and SM). For Likert-type items, frequencies and percentages were calculated to arrive at an understanding of the utility of the proposed app content for breaking barriers to professional help-seeking.

## Results

Sociodemographic profile of the participants

The sociodemographic details of the sample, as depicted in Table 1 and Table 2, show that both men and women were well represented in the study. The average participant in the D-TS group was 33 years old, while the average age of the D-NTS group was 23 years. The time between the realization of a need for help and first contact with mental health professionals/services was variable. About half of the D-TS group took more than one year to reach out to a professional. The D-NTS population reported that the duration of their current concerns ranged from a few months to several years. Moreover, 40% of the participants in this group reported not having ever thought of seeking professional help, while the rest (60%) reported having thought about the same at some point (Table 2).

**Table 1:** Characteristics of distressed treatment seekers SD: standard deviation

Variables	Frequency (n = 10)
Gender	
Male	6
Female	4
Age, years (mean age ±SD: 33.45 ±9.95 years)	
17-25	2
26-35	3
35-50	5
The time interval between the realization of a need for help and the first contact with a mental health professional	
Less than a month	4
6-8 months	1
More than 12 months	5

**Table 2:** Characteristics of distressed non-treatment seekers SD: standard deviation

Variables	Frequency (n = 10)
Gender	
Male	5
Female	5
Age, years (mean age ±SD: 23.4 ±6.8 years)	
17-25	9
26-35	0
35-50	1
Thought of seeking professional help for current concerns at any point	
Yes	6
No	4

Barriers to seeking help from mental health professionals/services

The researchers explored perceived barriers and reasons for hesitation in reaching out to mental health professionals/services in sampled participants. The content analysis of the data obtained indicated that these could be grouped under seven major themes (Table 3), namely situational barriers, fear about social consequences, doubts about the nature of the treatment, self-reliance, lack of support to seek help, difficulties related to self-disclosure, and inadequate self-awareness about mental health problems. Situational barriers included responses that mentioned external barriers such as distance, time, and availability/accessibility issues in reaching out to mental health professionals, e.g., “high costs involved”, “not many professionals available”, and the like. Fear about social consequences summarized participant responses that indicated a fear of being stigmatized or labeled by others including family, friends, and colleagues. The doubts about the nature of the treatment category included responses that indicated inadequate knowledge or awareness about the treatment or its usefulness and concerns about therapy/medication. These treatment-related concerns may stem from insufficient awareness, misconceptions due to inaccurate depictions of mental health issues/treatment in the media, vicarious negative experiences of significant others, and the like. The self-reliance category consisted of responses that indicated that participants wanted to be self-sufficient and help themselves or were doubtful that others could help them (e.g., “I can do it myself”). Lack of support to seek help entailed responses that indicated that there was no support from people around the person to seek help, or they did not consider it a mental health problem (e.g., there is no awareness about mental disorders culturally or in society). Responses that reflected the anticipated difficulty in sharing emotional concerns with others, especially when talking about it to strangers (professionals) were grouped as difficulties related to self-disclosure (e.g., “I fear opening up to a stranger”). The final theme that emerged in the data was inadequate self-awareness about mental health problems.

**Table 3:** Perceived barriers in seeking mental health professional services D-TS: distressed treatment seekers; D-NTS: distressed non-treatment seekers

Emergent themes	Overall frequency (n = 20)	Overall percentage (%)	D-TS frequency (n = 10)	D-NTS frequency (n = 10)
Situational barriers	10	50	2	8
Financial constraints				
No transportation/accessibility				
Time-consuming process				
Lack of availability of services				
Fear about social consequences	10	50	6	4
Negative label/stigma				
Fear about family, friends, or colleagues’ reaction/response/judgment (change in behavior)				
Doubts about the nature of the treatment	10	50	5	5
Lack of knowledge or awareness about treatment				
Doubts about the usefulness of therapy				
Concern about finding the right professional				
Negative experience with counseling/therapist				
Self-reliance	8	40	4	4
I can/should help myself				
Don’t know if other people can help me				
Lack of support to seek help	5	25	3	2
Others do not consider it as a problem				
Difficulties related to self-disclosure	3	15	2	1
Too emotional to talk to others about mental health				
Not comfortable to talk to strangers about my personal matters				
Inadequate self-awareness about mental health problems	5	25	4	1
Unsure if the problem is significant				
It is just a passing phase				
It is understandable given the situation				

The study also explored barriers faced during the actual utilization of mental health services, as reported by distressed treatment seekers (Table 4). Practical barriers that posed challenges in the continuous utilization of mental health services included time, financial constraints, and accessibility issues in terms of distance and appointment difficulties. Treatment seekers also reported some personal barriers during the utilization of mental health services. These included fears about the permanence of the mental health condition, side effects of medication, and dissatisfaction related to interactions with the treating professionals.

**Table 4:** Experience of barriers during the utilization of mental health services by distressed treatment seekers

Barriers	Frequency (n = 10)
Time-consuming process	1
High costs involved	3
Incorrect diagnosis/lack of expertise	2
Fear about the side effects of medication	3
Fear that it is a permanent condition	1
Difficulty in getting an appointment	2
Characteristics/attitude of the professionals (not giving enough time, asking “why?”, giving lots of therapy-related homework)	3
Difficulty in accessibility	1

Enablers in seeking help from mental health professional/services

Enablers refer to any process/factors that facilitate individuals to seek help from a professional. We have provided a summary of enablers reported by both groups in the present study (Table 5). Severity and duration of distress and associated recognition of need (50%), and support from family/friends (45%) emerged as the top two enablers for seeking professional help. Enablers for continued utilization of mental health services by current treatment seekers were also explored in the study. These included improvements observed during therapy, engagement in group sessions, and financial assistance in the form of some insurance coverage.

**Table 5:** Perceived enablers/motivators to seek mental health professionals/services D-TS: distressed treatment seekers; D-NTS: distressed non-treatment seekers

Themes	Overall frequency (n = 20)	Overall percentage (%)	D-TS frequency (n = 10)	D-NTS frequency (n = 10)
Level and duration of distress (perceived need)	10	50	8	2
Mental health literacy through others/social media/education	7	35	5	2
Support from family members/friends to seek treatment	9	45	6	3
Lack of informal sources of support	2	10	0	2
Belief in treatment/preventive measure	5	25	2	3

Perceived utility of proposed app contents for breaking barriers to seeking professional help

Participants were asked to rate the usefulness of the proposed app components in breaking barriers to seeking professional help (Table 6). Features such as information about symptoms, videos of client experiences, standardized questions and individualized feedback, videos by mental health professionals regarding therapy/other modes of treatment, experiences of clients in text/audio format, tips to overcome fears about medical and psychological treatments, visuals on facts or myths, online/video chat with experts, and a directory of professionals were rated as highly useful by more than 70% of all the participants.

**Table 6:** Perceived utility of the proposed content for overcoming barriers to seeking professional help D-TS: distressed treatment seekers; D-NTS: distressed non-treatment seekers

Proposed content components	Overall frequency (n = 20)	Overall percentage (%)	D-TS Frequency (n = 10)	D-NTS frequency (n = 10)
Information about symptoms				
0-1 (no/little utility)	1	5	0	1
2-3	2	10	1	1
4-5 (high utility)	17	85	9	8
Videos of client experiences				
0-1 (no/little utility)	1	5	1	0
2-3	1	5	0	1
4-5 (high utility)	18	90	9	9
Standardized questions about distress and individualized feedback				
0-1 (no/little utility)	0	0	0	0
2-3	0	0	0	0
4-5 (high utility)	20	100	10	10
Video of an expert about medical treatment/therapy				
0-1 (no/little utility)	0	0	0	0
2-3	2	10	0	2
4-5 (high utility)	18	90	10	8
Client testimonials in audio/text				
0-1 (no/little utility)	0	0	0	0
2-3	2	10	1	1
4-5 (high utility)	18	90	9	9
Tips to overcome fears about medical and psychological interventions/treatment for mental health concerns				
0-1 (no/little utility)	2	10	2	0
2-3	2	10	1	1
4-5 (high utility)	16	80	7	9
Visuals on facts or myths				
0-1 (no/little utility)	2	10	1	1
2-3	3	15	2	1
4-5 (high utility)	15	75	7	8
Identifying mental block/barriers to seeking help and individualized inputs/ feedback				
0-1 (no/little utility)	1	5	1	0
2-3	11	55	6	5
4-5 (high utility)	8	40	3	5
Online chat with an expert				
0-1 (no/little utility)	1	5	1	0
2-3	3	15	0	3
4-5 (high utility)	16	80	9	7
Directory of professionals				
0-1 (not at all/slightly useful)	3	15	3	0
2-3	0	0	0	0
4-5 (high utility)	17	85	7	10
Brief online contact with a professional				
0-1 (no/little utility)	3	15	3	0
2-3	0	0	0	0
4-5 (high utility)	17	85	7	10

## Discussion

Most of the participants were in their young and middle adulthood phases; this may be attributed to the prevalence of common mental health problems in this age group [1,13]. The higher proportion of young adults in the D-NTS group may partly be a methodological artifact as the recruitment of individuals for this group mainly relied on online platforms that have a wider reach and access for young adults [12,14]. However, it may also partly be attributable to the higher prevalence of D-NTS young adults in the community and delays in treatment-seeking by individuals experiencing common mental health concerns [1]. This can also be linked to a lack of awareness of the help available in the community for such problems. The pattern of findings in terms of delays in help-seeking implies the presence of various internal and external barriers to seeking help from mental health professionals/services in our sample of distressed urban Indian adults. According to the recent National Mental Health Survey (2015-2016), one in every 20 individuals in India is estimated to have clinical depression, and one in 25 is likely to be dealing with an anxiety disorder. Moreover, the treatment gap for mental disorders is as high as 86% [1]. Our study findings are in line with these observations.

On examining the broad themes related to barriers to help-seeking, it can be deduced that one can experience barriers at various points in the continuum of the help-seeking process. This can range from identifying if there is a problem in the first place to having concerns about from whom to seek help. These findings are in line with other studies that have highlighted the role of stigma, lack of easy access, low self-awareness, tendency to normalize, and excessive self-reliance as barriers to professional help-seeking [5,7,15,16]. Inadequate self-awareness was also perceived as a barrier by a quarter of the overall sample, apart from situational barriers, doubts about the nature of the treatment, fear about social consequences, and preference for self-reliance. This may stem from poor mental health literacy as applied to one’s concerns as well as participants’ difficulty in differentiating clinical problems from normal/understandable ups and downs in one’s mood and functioning as a result of life situations [17,18].

While comparing the nature of barriers reported by the D-TS vs. D-NTS groups, it was observed that doubts about the nature of treatment and excessive self-reliance were mentioned equally by both the groups as barriers. Inadequate self-awareness about mental health problems was reported as a barrier mostly by the D-TS group. On the other hand, situational barriers were reported most by the D-NTS group (Table 3). The fact that situational barriers were reported less frequently by the D-TS group makes intuitive sense because those who are already seeking treatment are likely to have overcome at least some of these practical constraints. Using Gulliford’s terminology, the D-TS group reported more personal barriers, while the D-NTS group reported more situational barriers [19]. As practical barriers appear to be a major constraint for the D-NTS group in the community, making mental health services easily accessible with judicious use of digital platforms may enable this group to reach out for professional help [20]. At least one study indicates that initial engagement with online professional help can work as an enabler for subsequent direct or face-to-face contact with mental health services [21]. It is also plausible that distressed individuals, particularly those who have not yet sought professional help, are not aware of or have difficulties in articulating their personal barriers towards professional help-seeking. On the other hand, D-TS may be better at realizing, in retrospect, that limited self-awareness caused a delay in their seeking professional help. The process of interacting with mental health professionals and receiving treatment may improve self-awareness about mental barriers experienced earlier. These possibilities need to be examined further in future studies.

Practical barriers were often reported by the D-TS group during the utilization of professional services in the present study. In line with this, concerns relating to time and money have been commonly reported in previous studies [5]. These indicate limitations in existing mental health services and highlight the need for making services more accessible and user-friendly. Additionally, personal barriers during service utilization such as fears about the permanence of mental health conditions and side effects were also reported by the D-TS group. This suggests a need for sustained engagement with users of outpatient mental health services in the form of psychoeducation. It also points toward the need to listen to client perspectives during service delivery. Also, it calls for a heightened focus on the quality of mental health service delivery, especially the interactional aspects of the delivery. Negative experiences with professionals can become a barrier to continuing treatment and reaching out for professional help in cases of future difficulty [22]. Previous studies have looked at affordability, accessibility, and acceptability for the utilization of mental health services [19]. The pattern of findings in the present study reiterates the importance of these factors.

There is a dearth of studies that examine facilitators for help-seeking from mental health professionals [23]. In the present study, apart from perceived need, support from family members/friends emerged as an important enabler to professional help-seeking. Family support and supportive interactions helped them to understand the emotional concerns that their family members hold, which motivated them further to seek help. These observations are in line with previous studies [24]. It is interesting to note that while support from family members/friends to seek professional help acted as a facilitator for 45% of the participants, in some cases, the lack of informal support pushed individuals to seek help from a professional. Thus, social support may act as a deterrent or facilitator for the decision about or the uptake of mental health services, depending on the need of the distressed individual and the type or quality of support available [25]. One participant reported wanting to talk to someone else as a facilitator, while for others, talking to a stranger was a perceived barrier. Mental health literacy and belief in treatment emerged as other enablers in the present sample. The D-TS group reported more facilitators/enablers to seek help than the D-NTS group. This may partially explain why the D-NTS group had not been able to overcome barriers and seek help.

The improvement observed in one’s health and contact with others who have similar conditions were reported to be facilitators of continued utilization of services. Contact with others who have the same condition, in direct or indirect ways, and group-based intervention can be useful for reducing stigma and decreasing a sense of isolation [26,27]. Availing services in a community-based well-being center rather than a crowded hospital setting was another factor that encouraged continued engagement with mental health services in the present sample. This community-based well-being center offers easy access to interventions, short waiting time in a pleasant, less crowded outpatient care environment. Moreover, its name implies a focus on well-being and recovery. All of these factors might contribute to higher user satisfaction, de-stigmatization, and reiterate the need for organization of mental health services in a user-friendly manner.

Digital platforms can be a useful means for reaching out to individuals who are unwilling to seek face-to-face help from mental health professionals due to situational and/or personal barriers [28]. Although there are thousands of mental health apps that have come up in the last decade or so, many of these are under-researched or not sufficiently evaluated [29]. Moreover, to the best of our knowledge, most of the available apps are aimed at enhancing awareness, offering self-help interventions, or assisting in symptom monitoring [12]. They do not directly target mental barriers to seeking professional help. Given this background, we wanted to consider potential app components that might help in overcoming barriers to seeking professional help. Stakeholder perspectives regarding these potential app components were explored in this study.

The overall findings suggest that almost all the proposed components were rated as useful for breaking barriers to help-seeking, at least to some extent, by most participants in both the groups. Using standardized questionnaires to offer individualized feedback to users about the severity of problems was rated as highly useful by all the participants in both the groups. This reflects the felt need for receiving objective feedback about one’s mental health concern to decide about seeking professional help [30]. Components related to tips on overcoming fears about treatment, identifying one’s mental barriers, directory of professionals, and opportunity for brief online contact with a professional were rated as useful by a higher proportion of the D-NTS group as compared to those who were already seeking treatment. These observations suggest that the incorporation of such components in the proposed app may be useful for breaking barriers to help-seeking for D-NTS.

This was an exploratory study that relied on one-to-one interviews as a data collection method. Some of the limitations of this study include a small sample, and the inclusion of only those with common mental health concerns, limiting the scope of the study. D-NTS were recruited based on self-reported depressive and anxiety symptoms. Accessing D-NTS from the community can be challenging, and the mode of recruitment (announcements soliciting participation) depended on their initiation of contact and readiness to participate. The focus of the study was on a comprehensive description of barriers to help-seeking experienced by D-NTS and D-TS rather than a comparison of the barriers between these groups. Although a few similarities and differences between these groups have been observed, these observations need to be treated as tentative in view of the fact that the groups were not matched on age. This is one of the few studies that have explored stakeholder perspectives on a predetermined set of potential components for a mental health app. Another strength of the study is that it explored perspectives of both distressed persons who have reached out for and currently availing professional help as well as those who have not yet sought professional help. This gives us a larger picture of the barriers and enablers operating along the continuum of professional help-seeking.

## Conclusions

Overall, the findings suggest that situational barriers, fear about social consequences, and doubts about the nature of treatment were the top three barriers. Given these observations, a digital platform would be useful in addressing the unmet needs of the distressed urban population in the community. Individualized feedback and various other proposed components of the app were perceived as likely to be useful in breaking barriers to seeking professional help. Future steps should involve incorporating such components in the mental health app under development and testing their effectiveness in breaking barriers to reaching out for professional help for common mental health concerns.
